# BRAF^V600E ^mutations in malignant melanoma are associated with increased expressions of BAALC

**DOI:** 10.1186/1477-3163-7-1

**Published:** 2008-07-16

**Authors:** David Schrama, Gunhild Keller, Roland Houben, Christian G Ziegler, Claudia S Vetter-Kauczok, Selma Ugurel, Jürgen C Becker

**Affiliations:** 1 Department of Dermatology, Julius-Maximilians University, Würzburg, Germany

## Abstract

**Bachground:**

Activating *BRAF *mutations are present in approximately 50% of melanomas. Although different downstream target genes of the most common mutant V600E have been identified, the contribution of activating *BRAF *mutations to malignant transformation needs further clarification.

**Methods:**

Microarray gene analysis was performed for human melanoma cell lines harboring BRAF^V600E ^mutations in comparison to cell lines without this mutation.

**Results:**

This analysis revealed a more than two fold down-regulation of 43 and an increase of 39 gene products. *BAALC *(*Brain and acute Leukaemia, cytoplasmatic*) was most prominently regulated, since it was up-regulated in mutated cell lines by a mean of 11.45. Real time PCR analyses with RNA from melanoma cell lines (n = 30) confirmed the *BRAF*-activation dependent up-regulation of *BAALC*.

**Conclusion:**

*BAALC*, which has been associated with cell dedifferentiation and migration, may function as a downstream effector of activating *BRAF *mutations during melanomagenesis.

## Background

Activating mutations of the protooncogene *BRAF *have been observed in approximately fifty percent of malignant melanomas [1]. The V600E mutant accounting for over ninety percent of these mutations, obviates the requirement for segment phosphorylation of the T599 and S602 residues which is essential for a regular activation of BRAF [2,3]. Thus, the BRAF^V600E ^mutation leads to a continuous stimulation of the MAP kinase cascade which results in a variety of cellular changes such as proliferation and dedifferentiation [4]. However, the role of activating *BRAF *mutations with respect to course and stage of melanoma is still not defined. On the one hand, comparable incidence of activating *BRAF *mutations in invasive cutaneous melanomas and benign melanocytic nevi indicate that *BRAF *mutations alone are insufficient to cause malignant transformation [2,3]. In addition, low frequency of *BRAF *mutations in radial growth phase melanomas, i.e. the early phase of melanoma progression, suggests a correlation with progression rather than initiation [5]. On the other hand, in patients with metastatic melanoma the presence of *BRAF *mutations is associated with a significantly poorer prognosis [6]. Moreover, introduction of BRAF^V600E ^in melanocytes rendered them tumorigenic in nude mice [7], while another group demonstrated that BRAF^V600E ^expression in human melanocytes induced cell senescence leading to cell cycle arrest. Hence, additional mutations have to occur to overcome this cell cycle arrest and make the cells tumorigenic [8]. Various genes have been identified as possible targets of the RAS/RAF/MAP kinase pathway. In this regard, microarray gene expression profiling allows measuring the expression of a large number of genes at the same time and thus providing a method for predicting the impact of oncogenes on the expression of possible down stream target genes. For example, microarray analyses of transfected murine embryonic fibroblasts with oncogene expressing adenoviruses helped to identify complex genetic alterations caused by genes such as *HRAS*, *MYC *and the *E2F *family [9]. Similarly, using standard cDNA microarray chips, gene expression signatures were reported for malignant melanoma cell lines harboring mutations in the *BRAF *gene when compared to wild type cell lines [10-12]. In this study, we used customized microarrays to identify additional target genes of the constitutively active MAPK pathway. The BRAF^V600E ^dependent expression of a newly identified potential effector gene, *BAALC (brain and acute leukaemia, cytoplasmatic) *was confirmed by real time PCR analyses in complimentary experiments in melanoma cell lines.

## Materials and methods

### Melanoma cell lines and cell culture

16 human malignant melanoma cell lines harbouring BRAF^V600E ^mutations as well as 9 melanoma cell lines with activating RAS mutations and 5 human malignant melanoma cell lines without mutations in these genomic sections were cultured in RPMI 1640 medium supplemented with 10% fetal calf serum (Table 1). Prior to RNA isolation the presence or absence of the V600E mutation was confirmed by direct sequencing PCR amplicons of *BRAF *exon 15.

**Table 1:** BRAF and RAS mutational status of the analyzed cell lines.

**Wild type BRAF/RAS^1^**	**BRAF mutated^2^**	**RAS mutated^3^**
MV3	FM88 (V600K)	MaMel79 (Q61K)^5^
M19	IF-6 (V600E)	MaMel91 (Q61K)
MaMel2^4^	Mel2a (V600E)	MaMel28 (Q61R)
MaMel71	FM55 (V600E)	MaMel26a (Q61R)
MaMel15	FM82 (V600E)	M26 (Q61R)
	MaMel19 (V600E)	FM79 (Q61L)
	MaMel83 (V600E)	BLM (Q61R)
	MaMel86a (V600E)	MaMel5 (Q61R)
	MaMel85 (V600E)	MaMel27 (G12D)
	MaMel13 (V600E)	
	MaMel63a (V600E)	
	MaMel6 (V600E)	
	MaMel7 (V600E)^5^	
	MaMel4 (V600E)	
	MaMel92 (V600E)	
	MaMel80a (V600E)^5^	

^1 ^Respective cell lines possess neither a BRAF nor a RAS mutation

^2 ^Listed cell lines are mutated for BRAF. The mutation is given in brackets.

^3 ^Listed cell lines are mutated for RAS. The mutation is given in brackets.

^4 ^the MaMel cell lines are short term biopsy-derived cell lines demonstrating strong concordance with the mutational status of the corresponding tissues [24].

^5 ^Cell lines are hemi- or homozygote for the respective mutation.

### Gene expression analysis

Three melanoma cell lines (Mel2A, IF-6, FM88) with BRAF^V600E ^mutation and three without this mutation (M26, MV3, M19) were examined by cDNA microarray analyses as described elsewhere [13]. In brief, total cellular RNA was isolated with the RNAeasy kit (QIAGEN, Hilden, Germany) and a subsequent DNase digestion was included. Hybridization probes were generated by indirect labeling with Cy3 and Cy5 dyes, using the CyScribe cDNA Post Labeling Kit RPN5660 (Amersham Biosciences Europe, Freiburg, Germany). All procedures were performed according to the manufacturer's instructions with 60 μg of RNA. Each experiment was performed as sandwich hybridization, i.e. instead of a cover slip a second microarray slide was used. The 4.6 K cDNA chips were generated by the group of M. Krause [ (Research, Microarray Unit)] and contained the GF200 set of Research Genetics cDNAs. Fluorescence labeled cDNA was spotted in duplicates. A flip color experiment was included. Samples were hybridized to microarrays for 16 h at 55°C. Chips were scanned with a GMS 418 fluorescent scanner (MWG-Biotech), and the images were analyzed with IMAGENE 3.0 software.

### Quantitative RT-PCR analyses for BAALC

Relative expression of BAALC was determined by real time PCR analyses in Sybr green technology using the comparative ΔΔC_T _method. Total RNA was isolated from approximately 3 × 10^6 ^cells of human melanoma cell lines or from 25 six μm thick kryosections of melanoma tissue samples. Samples of total RNA were subjected to reverse transcription. Primers for *BAALC *were designed with Primer Express software (Applied Biosystems, Weiterstadt, Germany) and read as following: sense 5'-AGC-CGC-CGC-CAG-AGC-CGA-CAG-3'; antisense 5'-GG-GAT-CCA-GTG-CCG-TGA-AGG-3'. For the evaluation of BAALC expression the thermal cycling conditions comprised an initial denaturation step at 95°C for 10 min, then 43 cycles of three-step PCR including 94°C for 30 sec, 60°C for 30 sec and 72°C for 40 sec. GAPDH (Applied Biosystems) served as endogenous control. The relative expression level of BAALC, normalized to GAPDH and relative to the randomly selected human melanoma cell line FM79 (*BRAF *wt/wt) was calculated as $2^{-\Delta \Delta {\text{C}}_{\text{T}}}$ with ΔΔC_T _= (C_T BAALC, sample _- C_T GAPDH, sample_) - (C_T BAALC, FM79 _- C_T GAPDH, FM79_). C_T _is defined as the cycle when the threshold level of fluorescence is reached.

### Statistical analysis

In microarray analyses, the threshold value for up or down regulation of gene expression was defined as a more than two fold change compared to the mean value. Data of real time PCR studies were expressed as box-and-whiskers plot. Differences between the values were evaluated by Mann-Whitney test. p < 0.05 was considered to be significant.

## Results and Discussion

Activating mutations of *RAS *and *BRAF *result in a constant activation of the MAP kinase pathway and eventually contribute to proliferation and dedifferentiation of cancer cells [2,14]. Although numerous target genes of the RAS/BRAF MAP-kinase pathway have been identified so far, the mechanism of action by which activated BRAF contributes to the malignant transformation of melanoma cells needs to be further elucidated. Therefore, we investigated gene expression of human melanoma cell lines harboring activating BRAF mutations (Mel2A, IF-6, FM88) by cDNA microarray analyses and compared their gene expression patterns to melanoma cell lines devoid of mutations in this genomic section (M26, MV3, M19). To this end, In BRAF^V600E ^mutated cell lines a more than two fold decrease of 43 and increase of 39 gene products was detected (table 2). Due to the limited number of cell lines used in the microarray experiments each candidate revealed by this analysis should be confirmed in analysis of larger numbers of samples. Only such confirmation assays ascertain the respective genes to be effected by activated BRAF. Nevertheless, the most interesting gene found by these analyses was *BAALC (*brain and acute leukaemia, cytoplasmatic), since (i) it was highest up-regulated in all three cell lines harboring BRAF mutations by an average of 11.25 fold and (ii) it was not yet described by other groups analyzing the effect of BRAF mutation on gene expression in melanoma [10-12,15]. The latter, i.e. finding a prominent regulation of gene expression in one study but not in others, seems at first curious. However, it has been noticed that even when the same microarray chips are used, different results are obtained. The use of different expression pattern detection algorithms and lab-dependent differences were identified as source for such inconsistency. Lab-dependent differences include selection and treatment of samples to mRNA isolation, cDNA probe generation, chip hybridization conditions, chip lot and even the use of different chip scanners [16,17]. These findings sustain the notion that confirmation assays are mandatory in order to confirm different regulated expression for genes detected by microarray analysis.

**Table 2:** Changes of gene expression in V600E mutated melanoma cell lines compared to wild type melanoma cell lines as revealed by microarray gene expression profiling

**Increase of gene expression**			**Decrease of gene expression**		
**symbol**	**gene name**	**fold change**	**symbol**	**gene name**	**fold change**

SLC2A10	solute carrier family 2 (facilitated glucose transporter), member 10	3,64	SELK	selenoprotein K	2,14
ARG99	ARG99 protein	2,25	LPHN2	latrophilin 2	2,11
SOD3	superoxide dismutase 3, extracellular	3,71	ADRB2	adrenergic, beta-2-, receptor, surface	2,81
MAN1C1	mannosidase, alpha, class 1C, member 1	2,49	LOC51159	colon carcinoma related protein	2,56
DHX29	DEAH (Asp-Glu-Ala-His) box polypeptide 29	2,09	EGR2	early growth response 2 (Krox-20 homolog, Drosophila)	2,10
C20orf45	chromosome 20 open reading frame 45	2,70	BUB3	BUB3 budding uninhibited by benzimidazoles 3 homolog (yeast)	2,00
RPS6KA3	ribosomal protein S6 kinase, 90 kDa, polypeptide 3	3,07	VEZATIN	transmembrane protein vezatin	2,07
RGS1	regulator of G-protein signalling 1	4,62	SPIN	spindlin	2,36
COL4A2	collagen, type IV, alpha 2	3,09	STS	steroid sulfatase (microsomal), arylsulfatase C, isozyme S	2,74
ZCCHC6	zinc finger, CCHC domain containing 6	2,07	CDC2	cell division cycle 2, G1 to S and G2 to M	2,17
CNTN1	contactin 1	2,03	MT1B	metallothionein 1B (functional)	2,44
MGC13105	hypothetical protein MGC13105	2,03	PITX2	paired-like homeodomain transcription factor 2	2,35
SUHW2	suppressor of hairy wing homolog 2 (Drosophila)	2,48	PRKCG	protein kinase C, gamma	2,58
ICAM1	intercellular adhesion molecule 1 (CD54), human rhinovirus receptor	2,14	FABP5	fatty acid binding protein 5 (psoriasis-associated)	3,03
BAALC	brain and acute leukemia, cytoplasmic	11,45	EPHB1	EphB1	2,13
OSBPL8	oxysterol binding protein-like 8	2,13	EGR1	early growth response 1	2,97
RAB24	RAB24, member RAS oncogene family	2,19	PIGF	phosphatidylinositol glycan, class F	2,16
LOC54103	hypothetical protein LOC54103	2,16	DYRK3	dual-specificity tyrosine-(Y)-phosphorylation regulated kinase 3	2,55
HSPC195	hypothetical protein HSPC195	2,24	RANBP6	RAN binding protein 6	2,26
EHD3	EH-domain containing 3	2,56	C10orf36	chromosome 10 open reading frame 36	2,92
CFLAR	CASP8 and FADD-like apoptosis regulator	2,02	DHRS8	dehydrogenase/reductase (SDR family) member 8	2,05
ZFP28	zinc finger protein 28 homolog (mouse)	2,04	P8	p8 protein (candidate of metastasis 1)	2,08
GSTM3	glutathione S-transferase M3 (brain)	3,20	SCD4	stearoyl-CoA desaturase 4	3,23
PPP3R1	protein phosphatase 3 (formerly 2B), regulatory subunit B,	2,07	RAB27B	RAB27B, member RAS oncogene family	3,45
APMCF1	APMCF1 protein	2,18	ZFP36L1	zinc finger protein 36, C3H type-like 1	2,22
N33	Putative prostate cancer tumor suppressor	2,05	SLC2A3	solute carrier family 2 (facilitated glucose transporter), member 3	2,16
ALDH1A1	aldehyde dehydrogenase 1 family, member A1	5,29	THBS1	thrombospondin 1	2,07
NAP4	Nck, Ash and phospholipase C binding protein	2,13	FLJ90440	hypothetical protein FLJ90440	2,62
LZTS1	leucine zipper, putative tumor suppressor 1	2,00	C5orf13	chromosome 5 open reading frame 13	2,13
P2RY2	purinergic receptor P2Y, G-protein coupled, 2	2,11	MT1F	metallothionein 1F (functional)	2,10
APPBP1	amyloid beta precursor protein binding protein 1, 59 kDa	2,35	CAMK4	calcium/calmodulin-dependent protein kinase IV	2,07
SLC5A6	solute carrier family 5 (sodium-dependent vitamin transporter), member 6	2,18	DCT	dopachrome tautomerase (dopachrome delta-isomerase, tyrosine-related protein 2)	2,15
CITED1	Cbp/p300-interacting transactivator, with Glu/Asp-rich carboxy-terminal domain	2,67	EFG1	mitochondrial elongation factor G1	2,61
FYN	FYN oncogene related to SRC, FGR, YES	2,25	LDB2	LIM domain binding 2	2,24
FDFT1	farnesyl-diphosphate farnesyltransferase 1	2,42	COL9A3	collagen, type IX, alpha 3	4,45
GDF11	growth differentiation factor 11	2,44	BAIAP1	BAI1-associated protein 1	2,02
FOXO1A	forkhead box O1A (rhabdomyosarcoma)	2,38	ALDH1A3	aldehyde dehydrogenase 1 family, member A3	3,65
PSPC1	paraspeckle component 1	2,04	HSJ001348	cDNA for differentially expressed CO16 gene	3,79
CLCN5	chloride channel 5 (nephrolithiasis 2, X-linked, Dent disease)	2,10	IRAK1	interleukin-1 receptor-associated kinase 1	2,02
			NMNAT2	nicotinamide nucleotide adenylyltransferase 2	2,86
			MT1G	metallothionein 1G	2,98
			PRKCA	protein kinase C, alpha	2,27

The product of the *BAALC *gene has been previously discussed to be involved in cell dedifferentiation and motility. In differentiated cells *BAALC *is almost exclusively expressed in the central nervous system and in other neuroectodermal derived tissues. Moreover, BAALC expression has been described for CD34 positive hematopoietic progenitor cells from the bone marrow [18]. Notably, loss of expression of *BAALC *during differentiation of hematopoietic progenitor cells suggests that it might function in sustaining an undifferentiated state of these cells [19,20]. Interestingly, however, *BAALC *expression in both normal and malignant astrocytes is increased upon differentiation, suggesting complementary functions in different cell types [21]. Nevertheless, it has been postulated that a series of genes, frequently expressed in progenitor cells of the neuroectodermal and the haematopoietic system, maintain the proliferative capacity while inhibiting differentiation [22] and *BAALC *may belong to this group of genes. Recent studies performed in patients with leukaemic malignancies indeed suggest a role of *BAALC *in tumorigenesis. For example, blast cells from 28% of patients with acute myeloid leukaemia and 65% with acute lymphatic leukaemia displayed an overexpression of BAALC. In addition, high BAALC expression was identified as an independent risk factor in acute myeloid leukaemia [20]. The examination of 13 human tumor cell lines of non-hematopoietic origin, however, revealed BAALC expression in only five cell lines derived from glioblastoma while BAALC was not detectable in any of the other neoplastic cells indicating a specific function of BAALC in some tumors only [18]. Furthermore, the expression of BAALC in developing and mature muscle cells in mice suggests a possible role of BAALC in cell locomotion or adhesion [23].

**Figure 1:**
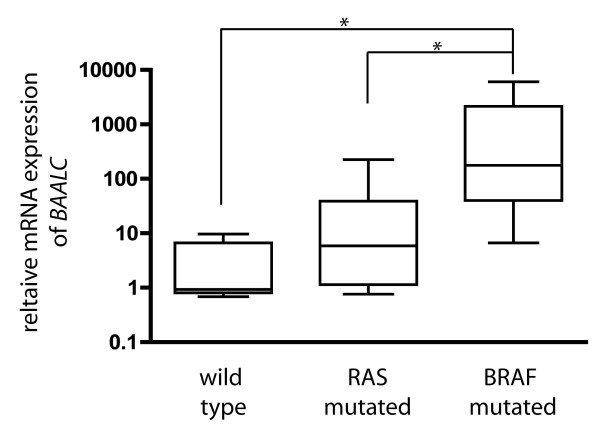
**Relative mRNA expression of *BAALC (brain and acute leukaemia, cytoplasmatic) *in human malignant melanoma cell lines as revealed by real time PCR**. 16 melanoma cell lines harboring BRAF mutation were compared to 9 cell lines with RAS mutation and 5 without any mutation in these genes. (* p < 0.01). As calibrator served a wild type melanoma cell line.

The distinct increase of *BAALC *expression in all three BRAF^V600E ^mutated melanoma cell lines suggests a possible function of BAALC associated with activated BRAF mutations in melanoma and mediating cell dedifferentiation and motility. As mentioned before, complementary experiments using real time PCR analyses to measure the relative mRNA expression of *BAALC *have to be performed to confirm this correlation. To this end, BAALC expression was determined in 16 cell lines mutated for BRAF, 5 wild type BRAF melanoma cell lines as well as 9 melanoma cell lines in which the MAP kinase pathway was activated by RAS mutations. These analyses validated the first observations; BRAF^V600E ^cell lines showed significantly (p < 0.01) elevated mRNA levels of *BAALC *(Fig. 1a) when compared to wild type cell lines. Notably, over-expression of *BAALC *was also significant when compared to RAS mutated cell lines (Fig. 1a). Importantly, most of the cell lines analyzed were short term cultures which should therefore resemble closely the parental tumor (table 1). Our results are in accordance with previous published data demonstrating that only a portion of regulated genes in cell lines with BRAF or NRAS mutations are common among the different mutations [10,11]. The different expression patterns might be ascribed to the differential capacity to receive input signals and to pass these on to various effectors.

In summary, BAALC may function as an additional mediator of activating BRAF mutations. Future studies will have to clarify its exact role in malignant transformation of melanocytic lesions.
